# Early Triassic Griesbachian microbial mounds in the Upper Yangtze Region, southwest China: Implications for biotic recovery from the latest Permian mass extinction

**DOI:** 10.1371/journal.pone.0201012

**Published:** 2018-08-08

**Authors:** Xiong Duan, Zhiqiang Shi, Yanlong Chen, Lan Chen, Bin Chen, Lijie Wang, Lu Han

**Affiliations:** 1 State Key Laboratory of Oil and Gas Reservoir Geology and Exploitation, Institute of Sedimentary Geology, Chengdu University of Technology, Chengdu, China; 2 Early Life Institute, State Key Laboratory of Continental Dynamics, Shaanxi Key Laboratory of Early Life and Environments, Northwest University, Xi’an, China; 3 Institute of Petroleum Engineering, Chongqing University of Science and Technology, Chongqing, China; Indiana University Bloomington, UNITED STATES

## Abstract

Early Triassic microbialites are distributed widely in the shallow marine facies of the Tethys Region, especially in the carbonate platform where they were deposited immediately after the latest Permian mass extinction (LPME). Ten Griesbachian domed microbial mounds were found in an outcrop of the uppermost first member of the Feixianguan (FXG) Formation at Baimiaozi, which is located in Beibei in the Upper Yangtze Region of southwest China. Field investigations and thin-sections analyses indicated that oolitic limestone, bioclastic limestone, microbialite, marl, and mudstone deposits are present in the first and second members of the FXG Formation, among which the thickness of the microbial mound above the massive oolitic limestone at the carbonate platform was approximately 3–4 m. Three facies were identified at the microbial mounds, namely, a mound base, mound body, and mound cap. Irregular laminae were found in the brown-colored microbialite of the mound base. The main mound body, which is composed of gray microbialite, is 1.0–1.5 m high and 2.0–3.0 m in diameter at the base. Dark gray grainstone found in the mound cap is covered by a thin layer of shelly limestone containing intact fossils of bivalves and gastropods, which are indicative of a simple ecosystem consisting of microbes and primary consumers. Brown-colored mudstone and marl layers of the second member of the FXG Formation overlie the microbialite, and this indicates that growth of the microbial mounds was halted by a sudden increase of terrestrial inputs and rapid transgression. Early Griesbachian conodonts of *Hindeodus parvus*? were identified from the mound limestone and the overlying strata of the second member of the FXG Formation, which is suggestive of the presence of a microbialite-dominated ocean in the Upper Yangtze Region during a certain interval after the LPME.

## 1. Introduction

The latest Permian mass extinction (LPME) (252.17 ± 0.06 Ma [1]) was the most prominent and severe event among the “big five” mass extinctions of the Phanerozoic [2, 3], and the subsequent biotic recovery took about 5 Ma [4–10]. This event caused the complete collapse of the ecosystems that had been established since the Ordovician, and it affected over 90% of marine species, 70% of land vertebrate genera, and most land vegetation [11]. The aftermath of the LPME was characterized by the expansion of microbialites into new environments that included offshore and nearshore ramps, platform interiors, and slope settings [12]. This expansion reflects the change in Late Permian oceanic conditions to lower O_2_ concentrations, higher HS^-^ contents, and anoxic waters that were supersaturated with respect to CaCO_3_ [12–16]. The deterioration of the oceanic environment caused the abrupt metazoan extinction, which was followed by the blooming of microorganisms during the earliest Triassic, when microbialite or microbial layers developed into more than 10 m thick deposits above the extinction boundary [2, 17–22].

The microbialites and microbes that were widespread above the Permian–Triassic boundary (PTB) are considered “unusual facies” or “disaster forms” [17, 23–26], which are characteristic of the substrate and ecological response during the period after the LPME, recording the decreased Early Triassic grazing and bioturbation pressures [12, 15]. Microbialites were deposited widely in the Precambrian ocean [27], and they declined after the metazoan blooms during the Ediacaran–Cambrian, especially after the great metazoan development [28]. Microbial activities blossomed during several episodes spanning 4 million years of the Early Triassic [22, 29] as a result of the harsh environment after the LPME, and microbialites became widespread in the shallow tropical ocean in the Early Triassic (summarized by [30]). Though some Early Triassic microbial mounds have been found at several sites, e.g., in the Nanpanjiang Basin in southern China and in eastern Greenland [31, 32], the importance of their temporal distribution, evolution, and effect on the slow metazoan recovery has not been completely elucidated. The isolated dome-shaped Smithian–Spathian microbial mounds in the Nanpanjiang Basin are about 1.5 m thick and are reported to be composed of a rigid calcimicrobial framework [31]. Furthermore, Wignall and Twitchett [32] described the existence of Griesbachian stromatolitic bioherms in eastern Greenland. Lower Griesbachian microbial mounds have been located at Baimiaozi (BMZ) in Beibei, southwest China (i.e., the eastern part of the Paleo-Tethys Ocean), and these are described in detail in the present study. The analysis of these microbial mounds is important for elucidating the changing environmental conditions and biotic recovery that occurred shortly after the Permian–Triassic (P–Tr) extinction event.

## 2. Geologic setting

**Fig 1:**
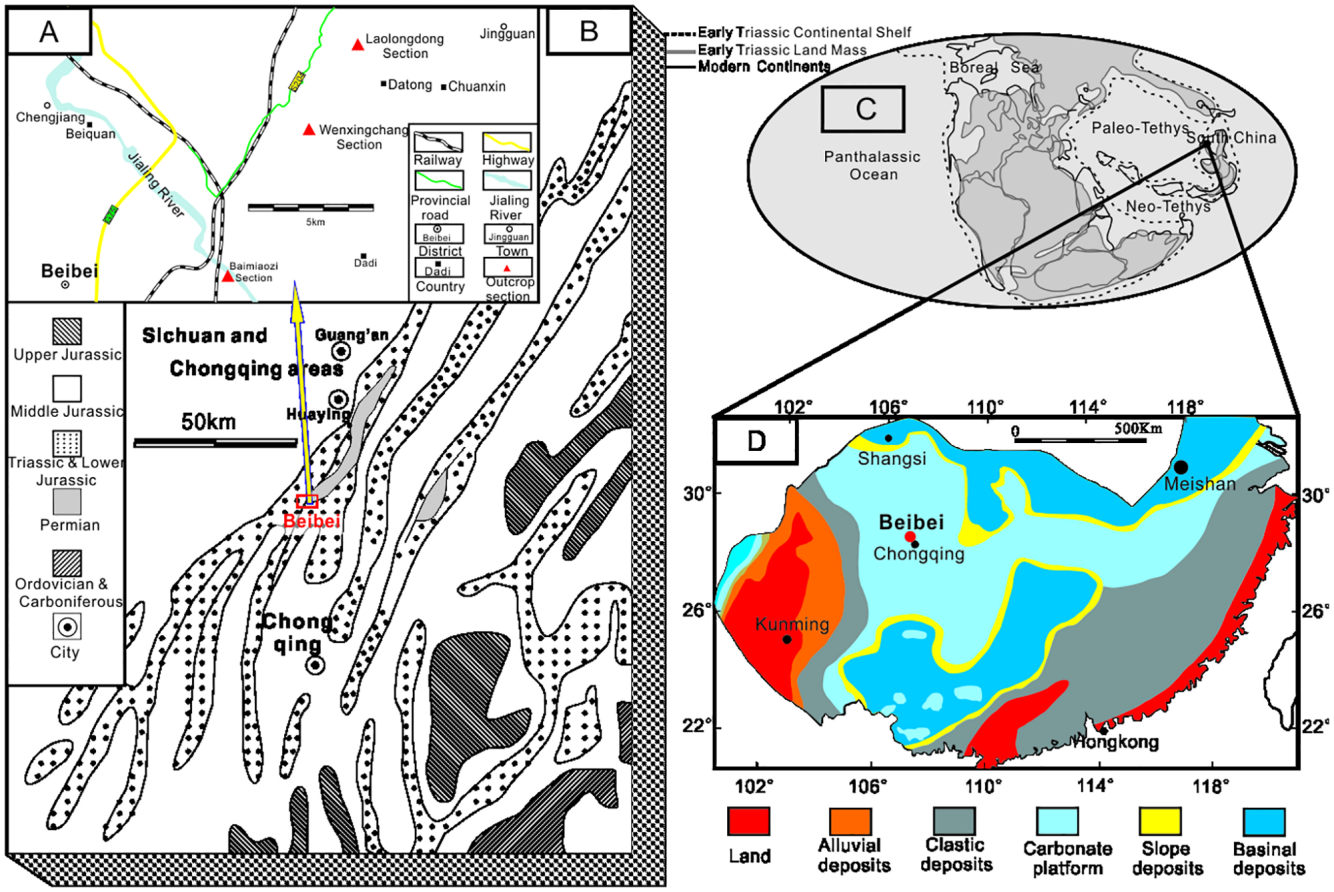
(A) Location of the Permian–Triassic boundary (PTB) sections in the Beibei area. (B) Location and regional geology of the study area (eastern Sichuan Basin), modified from [33]. (C) Global paleogeographic map, modified from [34]. (D) Early Triassic paleogeographic map of South China, modified from [35].

The well-exposed and successive Lower Triassic BMZ Section in Beibei lies along the Jialing River (29°48′43″N, 106°27′24″E), approximately 25 km to the north of the city of Chongqing (SW China) (Fig 1A). Geologically, this section is located at the Huayingshan Anticline Belt of the southeastern Sichuan Basin in the eastern Upper Yangtze Region (Fig 1B, modified from [33]). A large carbonate platform developed in the shallow sea of the Paleo-Tethys Ocean in the South China Block from the Late Permian to the Early Triassic (Fig 1C [34] and Fig 1D [35]), and many marine outcrop sections containing the carbonate platform sequence have been studied in an effort to reveal PTB events. Lithofacies paleogeography of the Late Permian and Early Triassic in southern China was controlled mainly by plate movement [36, 37]. The Early Triassic sedimentary and tectonic patterns were similar to those of the Late Permian, which were affected by the terrigenous detritus from the Kangdian Paleo-land and by the high production of carbonate in the shallow waters of the carbonate platform[36].

In the Huayingshan Anticline Belt, the latest Permian organic reefs (e.g., calcareous sponge reefs and coral reefs) and earliest Triassic microbialites have been widely reported on and studied [17, 33, 38, 39]. For example, Permian metazoan reefs and overlying microbialites have been found in the PTB transitional layers of the anticline belt at Laolongdong, Wenxingchang, Jianshuigou, and Dongwan, and these features are indicative of a change in the sedimentary environment and oceanic conditions during the P–Tr transitional interval [33, 38–43]. Small Late Permian patch reefs have been found in the Laolongdong Section, about 5 km north of the Beibei BMZ Section [44]. A transgression above the Permian reefs and two sea level drops have been identified above and below the PTB by Wu et al. [42] and Jiang et al. [45]. Shortly after the earliest Triassic sea level drop, a major transgression occurred [45], and a possible unconformity surface between the Permian patch reefs and the Griesbachian microbialites related to this event was reported by Kershaw et al. [41].

In the Chongqing area, volcanogenic clay beds found at the PTB sections [46] are considered to be the boundary of the Upper Permian Changxing Formation and the Lower Triassic Feixianguan (FXG) Formation. The FXG Formation, which is regarded as the Induan sequence by both Zhu et al. [47] and Huang et al. [48], is commonly divided into four lithological members containing two third-order cyclic sequences. The first member of the FXG Formation, which contains the bivalve *Claraia wangi* and the ammonoid *Lytophiceras* sp., is composed of mudstone in the lower part and of marl, micrite, and oolitic limestone in the upper part. Massive oolitic limestone (total thickness: >7.0 m) is distributed widely in the Beibei area according to the local geological maps produced by the Sichuan Bureau of Geology and Mineral Resources (1990–1992). The second member of the FXG Formation, which contains the bivalve *Claraia stachei*, is composed mainly of brown-colored mudstone and marl [47, 48].

In the BMZ Section yielding the microbial mounds from the south bank of Jialing River, however, the uppermost Permian limestone is not found and the oldest stratum seen in the core of the Huayingshan Anticline is lowest Triassic Dark brown shale. On the opposite side of the Jialing River, at a distance about 500 m away, the uppermost Permian bioclastic limestone can be distinguished from the core of same anticline (Fig 1A and 1B). Based on field investigations, we identified lower Triassic microbial mounds in the upper part of the first member of the FXG Formation at BMZ, Beibei. Conodonts from the upper first and lower second members of the FXG Formation suggest that the age of the microbial mounds is early Griesbachian.

## 3. Materials and methods

The present study was based mainly on an investigation of field outcrops, thin-sections observations, and conodont biostratigraphy. The succession of the upper first and lower second members of the FXG Formation at BMZ was examined, and the materials were photographed and sampled systematically. Samples were collected directly from the limestone of the microbial mounds. Twenty-four thin sections were prepared and photographed with a polarizing microscope (Nikon Lv100 pol) at the State Key Laboratory of Oil and Gas Reservoir Geology and Exploitation (Chengdu University of Technology). Our investigation focused on the microfacies of the microbial mounds and their upward change to the covered brown-colored shale of the second member of the FXG Formation.

**Fig 2:**
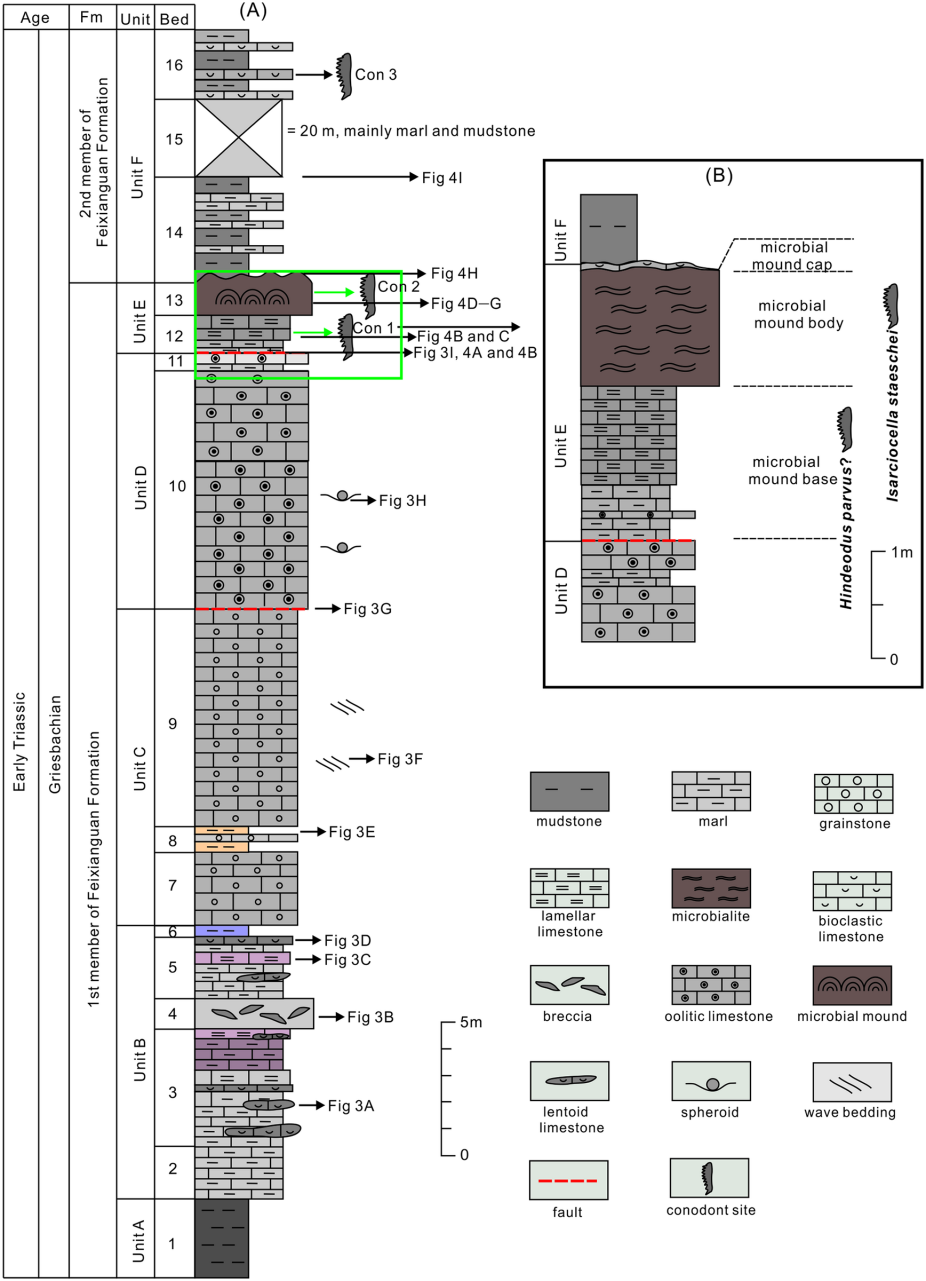
(A) General lithological column of the lower Triassic (Griesbachian) succession at the Baimiaozi Section in Beibei. (B) Lithological column of the investigated microbial mounds.

Ten conodont samples were collected from the ca. 50 m thick bioclastic and oolitic limestone of the first and second (lower part) members of the FXG Formation. The limestone samples were dissolved in 7% formic acid and then conodonts were separated using an LST heavy liquid (i.e., concentrated solution of lithium heteropolytungstates in water). The conodonts were photographed with a scanning electron microscope at the State Key Laboratory of Biogeology and Environmental Geology at China University of Geosciences (Wuhan). The conodont sample numbers (Con 1–3) are listed in Fig 2. Twenty-eight conodont fossils from the three rock samples collected from Baimiaozi Section, Beibei, Chongqing, are preserved as numbers of 100631, 100632, 100633 in the 15^th^ sample cabinet at the Museum of Institute of Sedimentary Geology, Chengdu University of Technology. The digging and site access permits of these rock samples are not required for this study.

Complied with Chinese relevant regulations, no permits are required for the described studying. The individual in this manuscript has given written informed consent to publish these case details

## 4. Results

### 4.1. Stratigraphy and facies

#### 4.1.1. General stratigraphy

The conformable boundary of the first/second members of the FXG Formation at the BMZ Section is characterized by a lithological change from gray massive limestone to dark brown mudstone. In this study, the first (lowest) member of the FXG Formation (outcrop thickness: approximately 36.0 m) was classified into five units and 13 beds. The lithological stratigraphy of the five units (Units A–E) of the first member of the FXG Formation and the lower unit of the second member of the FXG Formation (Unit F) is illustrated in Fig 2A and described below.

**Fig 3:**
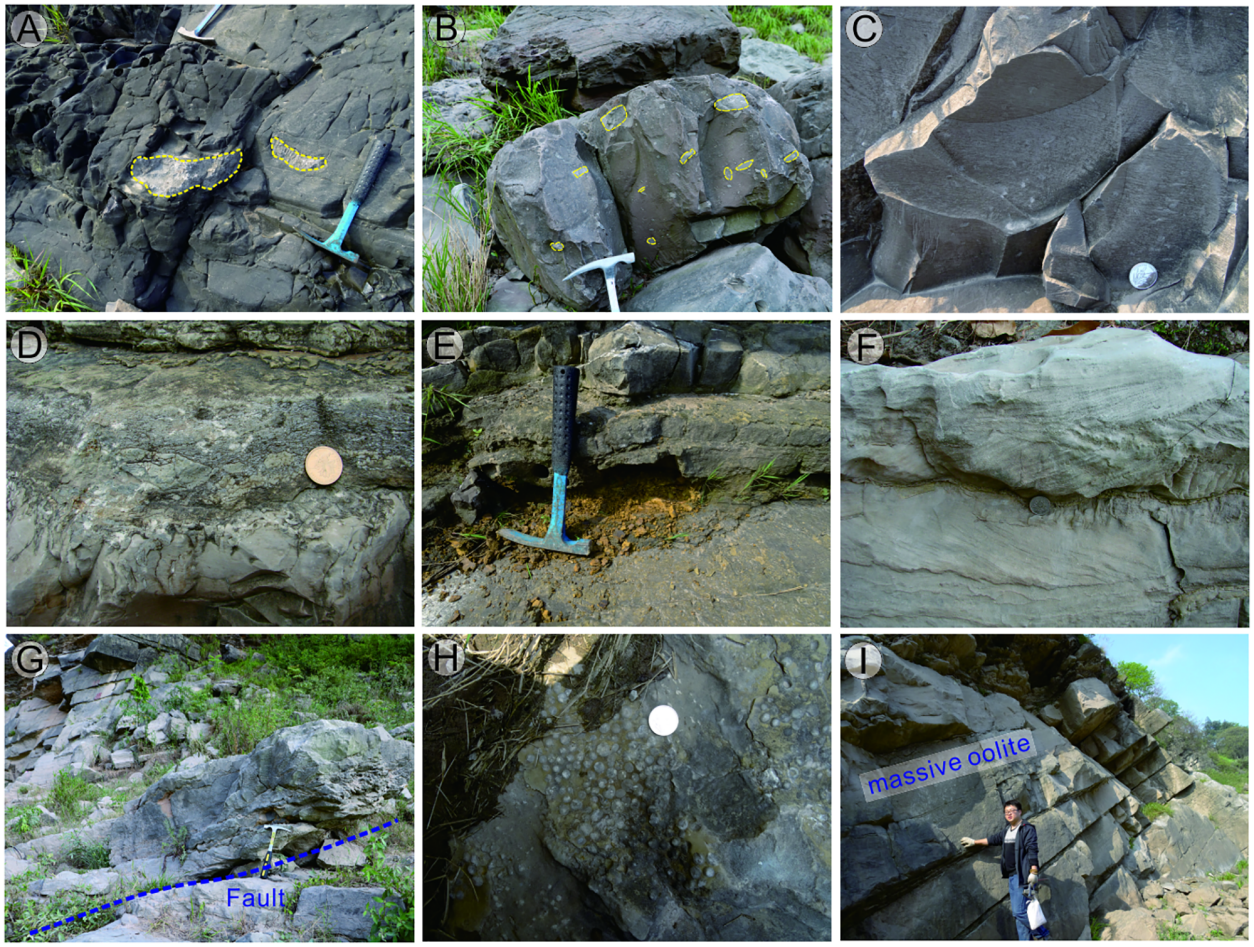
Outcrop images from the first member of the FXG Formation in the Baimiaozi Section. The locations of the pictures are shown in Fig 2. (A) Lenticular bioclastic limestone found in the massive marl layer of lower Unit B. (B) Breccia in the marl of Unit B. (C) Light purple lamellar limestone containing the spots of recrystallized calcite in Unit B. (D) Bioclastic limestone containing breccia, interlayered by marl in Unit B. (E) Yellow claystone found between the grainstone layers in Unit C. (F) Grainstone with wave-caused crossbedding and stylolite structure in Unit C. (G) Boundary (fault) between the grainstone of Unit C and oolite of Unit D. (H) Spheroids in Unit D yielded from the mudstone deposited in the wave trough of the top surface of the massive oolitic limestone. (I) Massive and thick bedded oolite in the upper part of Unit D.

**Fig 4:**
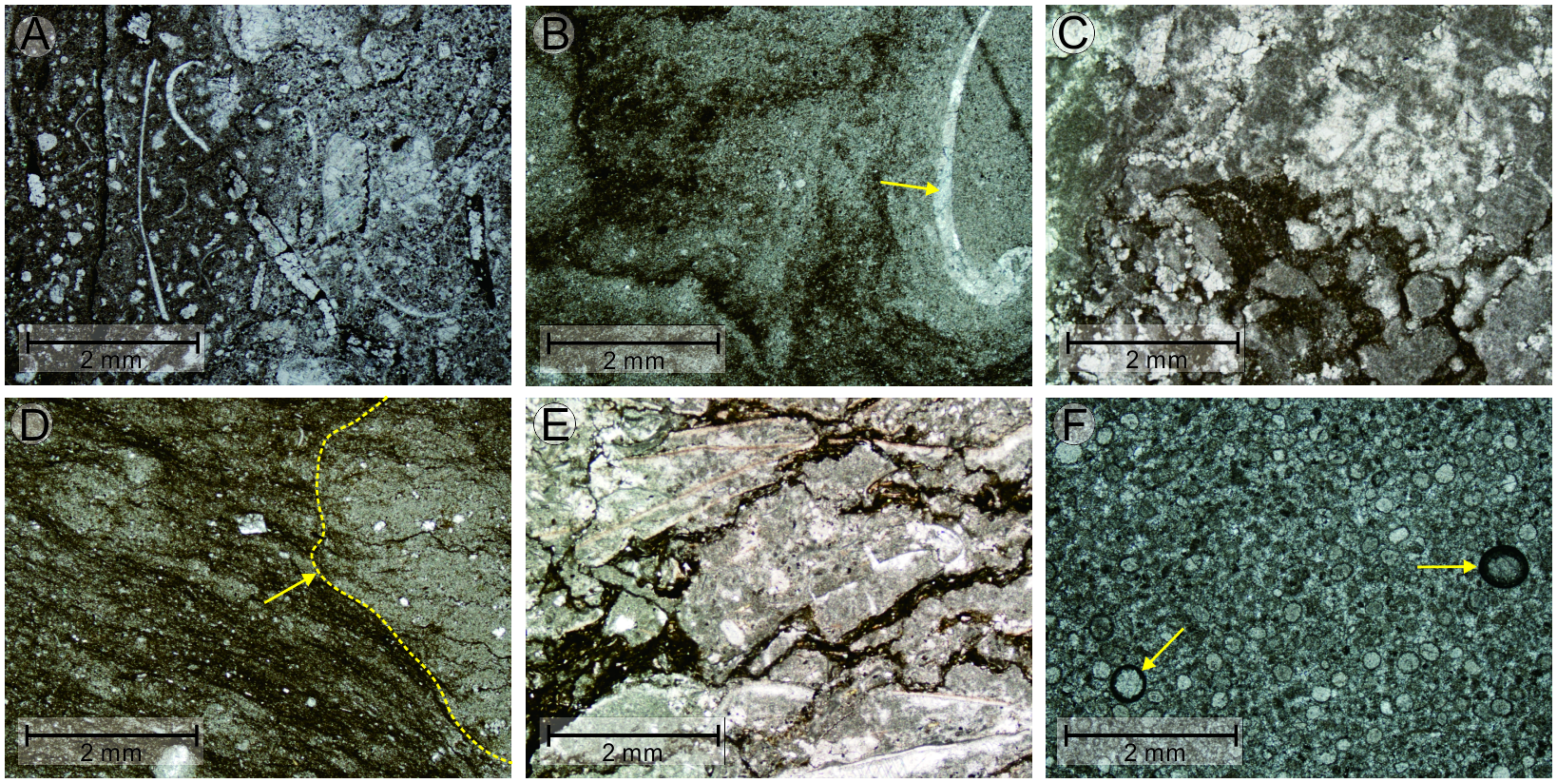
Thin-section microphotographs from the first member of the FXG Formation in the Baimiaozi Section. (A) Bivalve shell fossils are located mainly in the lenticular bioclastic limestone interlayered in the marl layers of lower Unit B. (B) Microphotograph of lamellar limestone showing impregnated clay material; the outcrop spot is composed of bivalve fossils and the is surrounded by recrystallized calcite (indicated by the yellow arrow) in Unit B. (C) Another thin section showing the light purple lamellar limestone of Unit B in which strongly recrystallized calcites are common. (D) Breccia with an irregular edge (indicated by the yellow arrow) found in the marl in Unit B. (E) Thin section of bioclastic limestone containing many stylolites in upper Unit B. (F) Peloids and recrystallized ooids surrounded by a micritization enclosure (indicated by the yellow arrows) in the oolite of Bed 10, Unit D.

**Fig 5:**
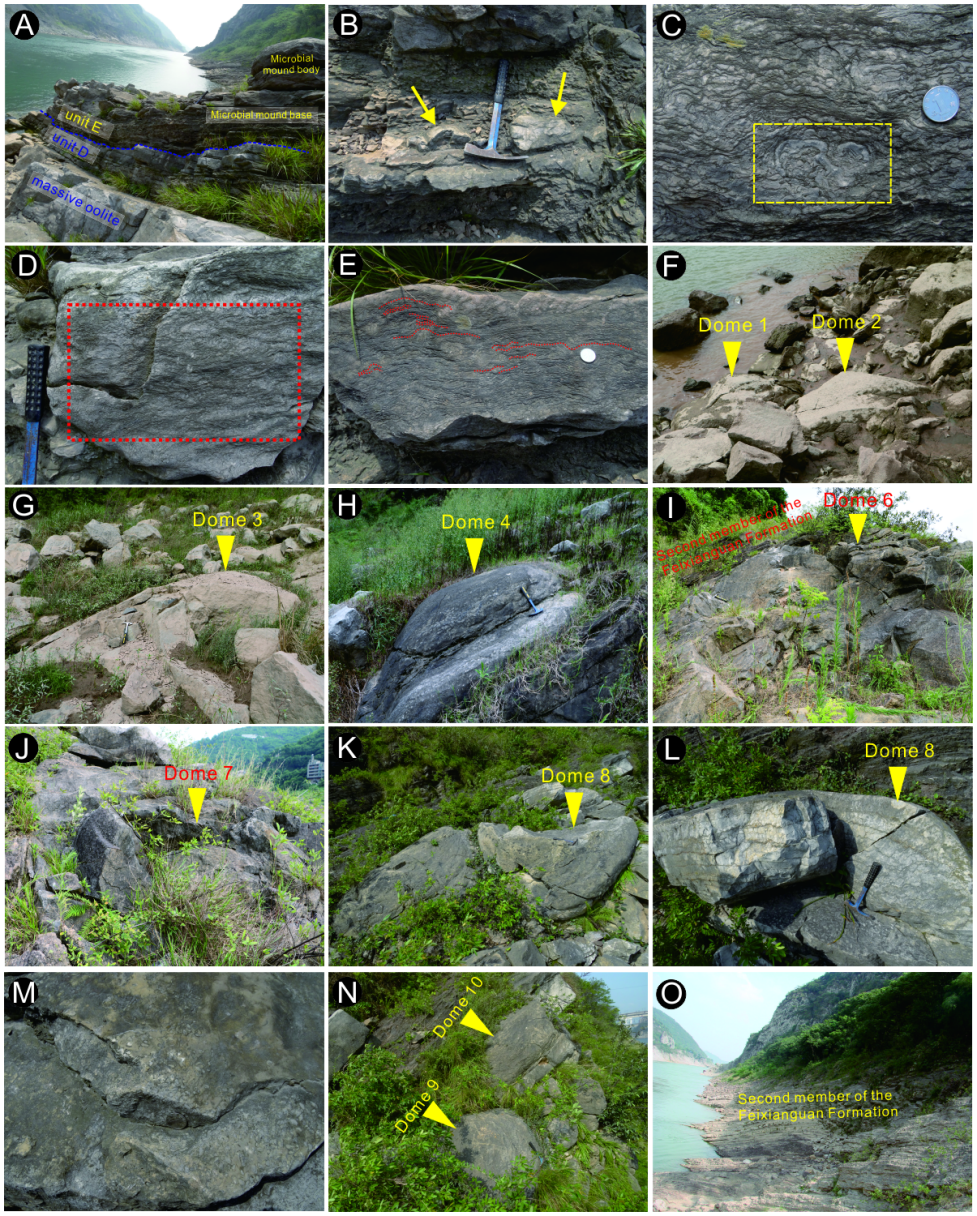
Outcrop images from the upper part (Units D and E) of the first and the lower second members of the FXG Formation. (A) The complete sequence of upper Unit D and Unit E located at the footwall of the reverse fault. (B) Interbedded lamellar limestone (microbialite) showing aborted microbial mounds and mudstone of the microbial mound base. (C) A rudiment of a microbial mound within the box defined by the dashed yellow line in Bed 12. (D) Horizontal structures of microbialites within the box defined by the dashed red line in Bed 12. (E) Wavy structures of microbialites in Bed 12. (F) Image of Dome 1 and 2 in the outcrop. (G) Image of Dome 3 in the outcrop. (H) Image of Dome 4 in the outcrop. (I) Gray mound body of Dome 6, and the overlying marl and mudstone of the lower second member of the FXG Formation. (J) Image of Dome 7 in the outcrop. (K) Image of Dome 8 in the outcrop. (L) Net-like stylolites developed in Dome 8. (M) Shelly fossils on the surface of the bioclastic limestone of the mound cap of Dome 8. (N) Image of Dome 9 and 10 in the outcrop. (O) Photograph showing the lower part of the second member of the FXG Formation.

1. Unit A (Bed 1): The outcrop is about 6.0–m thick and comprised chiefly of dark purple or brown-colored mudstone representing the lowest Triassic sequence deposited shortly after the PTB event. This unit lacks marine metazoan fossils.
2. Unit B (Beds 2–6): This unit contains an approximately 11.0 m thick interval of light purple lamellar limestone (Fig 3C), marl (Fig 3A and 3B), and gray lentoid bioclastic limestone interbeds (Figs 2 and 3D). In the lamellar limestone, irregular laminae develop as horizontal and wavy structures and Wu et al. [49] interpreted the similar structures were microbiogenic. In Bed 4, the marl contains massive gray breccia of lentoid bioclastic limestone and micrite (Figs 2, 3A and 3B), which has been interpreted as seismites by Luo et al. [50]. Irregular laminae dominate in the lamellar limestone (Fig 3C), while impregnated clay material with outcrop spots of bivalve fossils (Fig 4B) and strongly recrystallized calcites (Fig 4C) can be seen in the microphotographs of these lamellar limestone layers. Bivalve fossils are contained predominantly in the lentoid or bedded bioclastic limestone (Fig 4A and 4E).
3. Unit C (Beds 7–9). This unit is 17.5 m thick and composed mainly of grainstone containing several main grain types such as bioclasts, intraclasts, peloids, and ooids. The main sedimentary structures seen in this unit include both wave and herringbone crossbedding (Fig 3F), which is suggestive of a sedimentary environment dominated by waves and tides.
4. Unit D (Beds 10–11). This unit is oolite with a thickness of ca. 7.0 m. A fault separates the grainstone of Unit C and the oolite of Unit D (Fig 3G), and dense fractures and calcite veins can be seen from the bottom of Unit D. Wave marks are common at the top surface of the massive or thick-bedded oolitic limestone that dominates Unit D. Spheroids, reported first by Pruss and Payne [51] from the Spathian limestone of the Moenkopi Formation in the western United States, can be found in the mudstone deposited in the wave trough at the top surface of the massive oolite (Fig 3H). Inside the oolite, the interstitial material between the ooids is commonly a micrite matrix (Fig 4F). The content of grains in the massive oolitic limestone is about 65%, and the interstitial substance (cement and matrix) comprises 35%. The thickness of the oolite layers decreases to 5.0–20.0 cm in the uppermost part of Unit D (Fig 5A).
5. Unit E (Beds 12–13). This unit is about 3.0-m thick and is characterized by chocolate marl, lamellar limestone, and gray microbial mound limestone (Fig 5F–5I). Overall, 10 mounds (Fig 5F–5N, Table 1) could be distinguished from the surface of this unit, covered by a layer of light gray bioclastic limestone with a thickness of approximately 5.0–12.0 cm (Fig 5M). The sizes of the 10 mounds are shown in Table 1. Conodont fossils such as *Hindeodus parvus*? and *Isarcicella staeschei* were found in the microbial mounds, and these fossils indicate that the age is that of the lower Griesbachian Stage. Only from Mound 6 and 8 can we see the integrated and clear internal structure of mound body and cap (Fig 5I and 5K). The detailed lithological and facies descriptions of this unit are presented in Section 4.2.
  A local reverse fault influenced the sequence of Unit E and caused a duplication of the stratigraphy from upper Unit D to Unit E. The fault displacement was estimated to be 4.0–5.0 m according to the field investigation.
6. Unit F (Beds 14–16). This unit contains the sequence of the lower part of the second member of the FXG Formation (Figs 2 and 5O) overlying the microbial mounds (Fig 5I, 5L and 5M). The thickness of this unit is >28.0 m, and the lithology is mainly brown-colored shale interlayered with gray marl and bioclastic limestones (Fig 2). Griesbachian conodont fossils were discovered in the thinly bedded bioclastic limestones of Bed 16.

**Table 1:** Sizes and characteristics of the microbial mounds at BMZ.

Dome	Site	Size	Characteristic	Note
Dome 1 and Dome 2	Riverside	Diameter 2.0–2.5 m, Height 0.7–0.8 m	Relatively small circular mounds	Fig 5F
Dome 3	Above Dome 2	4.0 × 7.5 × 1.5 (height) m^3^	Bigger than Dome 1 and Dome 2	Fig 5G
Dome 4	Above Dome 3	3.0 × 4.0 × 1.6 (height) m^3^	Regular domical mound	Fig 5H
Dome 5 and Dome 6	Above Dome 4	3.5–4.0 m (diameters) × 7.0 m (total length of composite mound) × 1.5 m (height)	These two mounds form a composite mound	Fig 5I
Dome 7	Above Dome 6	4.0 × 6.0 × >1.0 (height) m^3^	Composed mostly of branching and stromatolitic aggregates	Fig 5J
Dome 8	Above Dome 7	3.0 × 3.0 × >1.0 (height) m^3^	Net-like stylolite structures	Fig 5K
Dome 9	Beside Dome 8	>3.0 × 5.0 × >1.0 (height) m^3^	Affected by the fault	Fig 5N
Dome 10	Highest site	4.0 × 4.0 × 1.0 (height) m^3^	Affected by the fault	Fig 5N

#### 4.1.2. Facies of microbial mounds

James and Bourque [52] defined a microbial mound as an accretion of stromatolites or thrombolites, calcimicrobes, mud, and possibly other material. The depositional environment of microbial mounds should be a shallow sea with low energy [52]. Two prerequisites for defining a microbial mound are (1) a mound-like or lens-like shape different from the usually bedded surrounding rocks, and (2) the participation of microbes in the process of its growth [52, 53]. At least 10 microbial mounds exposed at the BMZ Section have the typical characteristics mentioned above (Fig 5F–5N). These mounds are commonly 1.0–2.0 m in height and extend 3.0–7.0 m laterally (Table 1). In reference to the reef facies described by Pomar [54], Álvaro and Debrenne [55], and Adachi et al. [56], the microbial mounds in this study can be considered to be composed of three facies, namely, a mound base, mound body, and mound cap.

**Fig 6:**
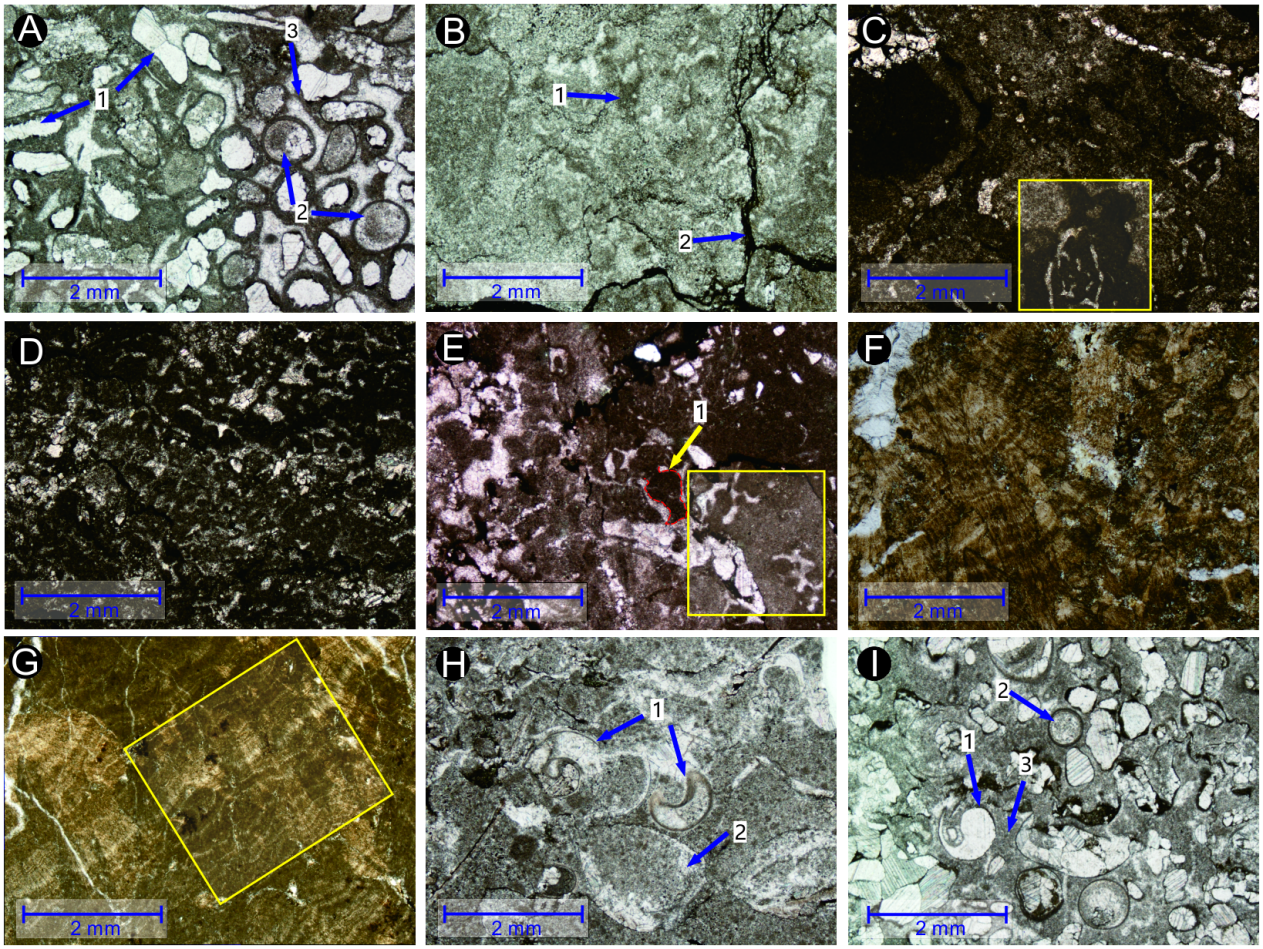
Thin-section photographs showing internal fabrics of microbial mounds. (A) Thin-bedded oolitic limestone of mound base facies in Bed 12 containing (1) recrystallized bioclasts, (2) ooids wrapped within a micrite envelope, and (3) the micritic matrix. (B) Microbialite (i.e., lamellar limestone) of mound base in Bed 12 showing (1) irregular micrite lumps and (2) stylolites. (C–E) The cores of the mound bodies in Dome 6 and Dome 8. (F) Microbialite in the middle of the microbial mound body in Dome 7 showing the branching structure of microbial aggregates. (G) Stromatolitic structures in Dome 7. (H) Bioclastic limestone of mound cap facies in uppermost Bed 13 showing (1) gastropod shells and (2) ostracod fossils. (I) Bioclastic limestone of microbial mound cap in uppermost Bed 13 at Dome 6 containing (1) gastropods, (2) ooids, and (3) the micritic matrix.

**4.1.2.1. Microbial mound base**. At BMZ, all the microbial mounds share the same mound base, which consist mainly of mudstone, thin-bedded oolitic limestone, and laminated limestone, and these materials are located above the medium-bedded gray oolite of the upper first member of the FXG Formation (Fig 5A). The lower part, i.e., the microbial mound base facies, is about 0.5 m thick and it comprises interbedded brown-colored mudstone, two interbeds of gray, thin-bedded oolitic limestone, and brown-colored lamellar limestone (Fig 5B). From the massive to the medium-bedded oolite of Unit D, and then to the thin-bedded oolite in the lower Unit E, the thicknesses of the oolite layers decrease (Fig 5A). Moreover, in comparison with the underlying massive oolitic limestone, the grain size of the mound base oolitic limestone is smaller and the average size of these ooids is about 0.3 mm. These two thin-bedded oolitic limestones consist mainly of ooids, intraclasts, peloids, sparry calcite cement, and a micritic matrix (Fig 6A). The content of grains in the thin-bedded oolitic limestone is about 70%, and the content of the interstitial substance (cement and matrix) is approximately 30%. In addition, microsparry calcite is also present, which might have formed during the recrystallization of the micritic matrix; these findings are suggestive of a hydrodynamically relative lower energy environment than that in which the underlying massive oolite formed. The interbedded lamellar limestone (about 15.0 cm thick) contains small microbial mounds with heights of 5.0–8.0 cm (Fig 5B). These are interpreted as aborted mounds whose growth was hindered by the deposition of superfluous inputs of terrigenous clay minerals.

The upper part of the microbial mound base is composed mainly of chocolate microbialite (i.e., lamellar limestone). Irregular laminae are commonly seen in these microbialites and the structures of these laminas can be divided into three types, namely, approximately mound-shaped (Fig 5C), horizontal (Fig 5D) and wavy (Fig 5E) structures. The mound structures are about 10.0–15.0 cm high and these may be interpreted as the rudiments of microbial mounds (Fig 5C) in accordance with its morphological features. Stylolites were found commonly (Fig 6B). The brown-colored microbialites consist mainly of microsparry calcite, micrite and occasional spar calcite, which shows a certain degree of recrystallization (Fig 6B). Furthermore, some irregular lumps are interpreted as being caused by microbial action within the microbialite (Fig 6B). In the thin oolitic limestone of the lower mound base, some carbonate grains are wrapped by micrite envelopes (Fig 6A), which also is indicative of contemporaneous microbial action. Microbialites containing approximately horizontal and wavy laminar structures are similarly composed mainly of microsparry calcite and micrite, although bioclastic grains can be rarely observed.

**4.1.2.2. Microbial mound body**. The microbial mound bodies are 1.0–1.5 m thick, and they are characterized by dome-shaped gray microbialites (Fig 5F–5N). Net-like stylolite structures are common in the mound bodies, e.g., Domes 3, 6, and 8 (Fig 5L); however, lumpy microbialites can be seen in Dome 7 (Fig 5J), and the microstructures of these two types of microbial mounds are different. The net-like stylolite structures consist mainly of intraclasts, bioclastic grains, peloids, and a micrite matrix (Fig 6C–6E). According to the structures shown in Fig 6D and 6E, the microbialites may belong to thrombolites which are depositions of cyanobacterial colonies ([57]). The bioclastic grains are mainly microscopic broken shell fossils (lengths: <1.0 mm) wrapped by a micrite envelope. Possibly, most of the grains and micrite have been recrystallized. In addition, in the stylolite structures, argillaceous laminated structures and irregular lumps can be observed. On the contrary, the lumpy microbialites (Fig 5J) are composed mostly of branching and stromatolitic aggregates (Fig 6F and 6G) and lack stylolites. Successive direct sampling from Domes 6 and 8 revealed little variation in their interior structures from the central to limbic parts of the mounds with net-like stylolite structures. The two types of microbialite mounds are therefore interpreted as the result of two types of microbial activity.

**4.1.2.3. Microbial mound cap**. The thin microbial mound cap (about 2.0–6.0 cm thick) is mainly gray or brown-colored biologic limestone. Abundant and well-preserved ostracod, bivalve, and gastropod fossils can be seen on the wavy surface (Fig 5M). The sizes of these fossils range from 1.0–2.0 mm to 3.0 cm. Microscopically, the biologic limestone consists mainly of complete marine fossils, peloids, and a micrite matrix, although a certain amount (about 25%–30%) of the fossils and micrite matrix have been recrystallized (Fig 6H and 6I). Some grains (e.g., ooids and bioclasts) are wrapped by a micrite envelope (Fig 6I). Moreover, small amounts of ooids and calcite intraclasts occur within the biologic limestone of the mound cap (Fig 6I).

### 4.2. Conodont biostratigraphy

**Fig 7:**
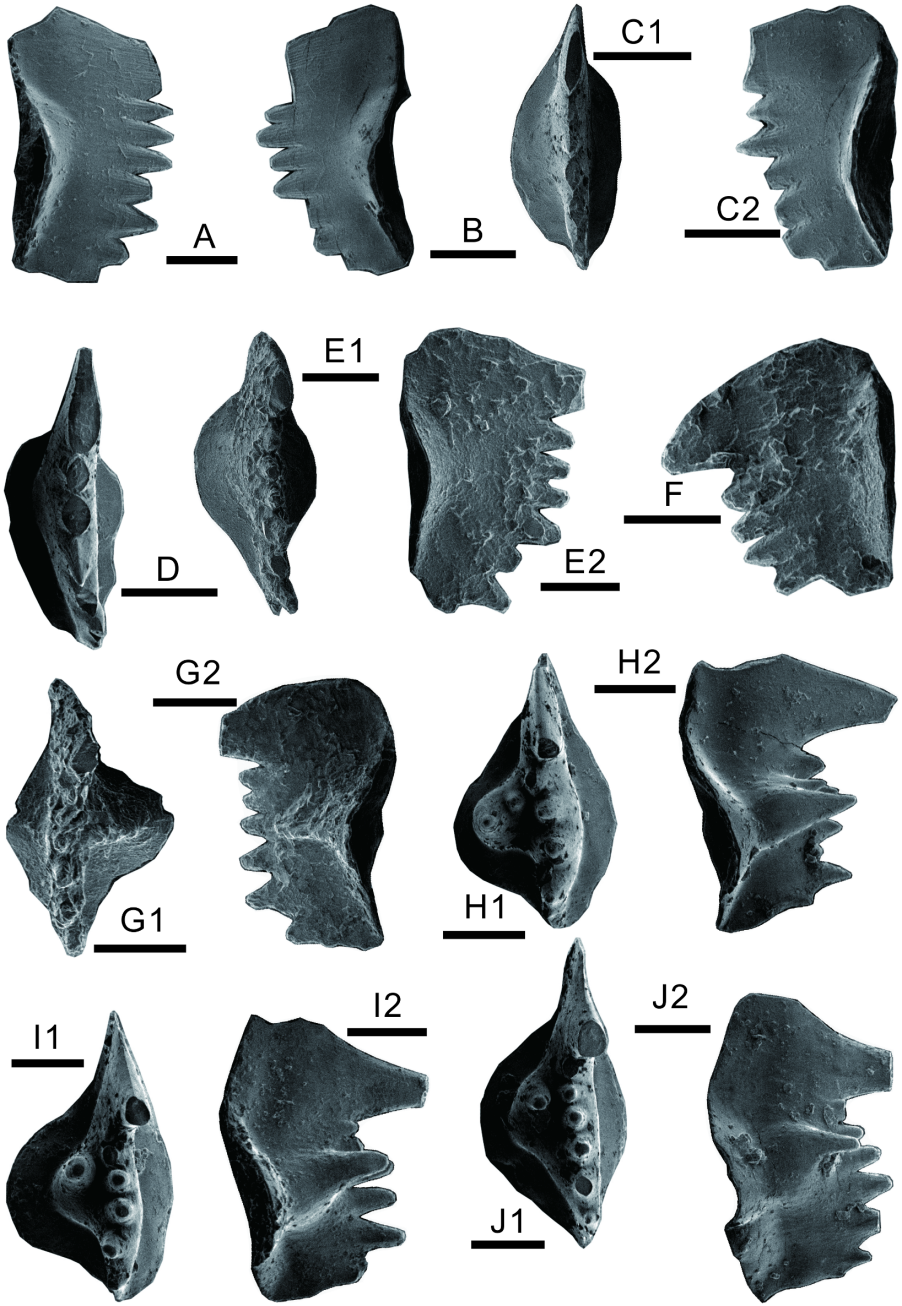
Conodonts from the lower Triassic microbial mounds at Baimiaozi, Beibei. A–F, *Hindeodus cf*. *parvus* (Kouzr and Pjatakova, 1976). A, lateral view; B, lateral view; C1, upper view; C2, lateral view; D, upper view; E1, upper view; E2, lateral view; F, lateral view. G–J, *Isarclcella staeschei* Dai and Zhang, 1989. G1, upper view; G2, lateral view; H1, upper view; H2, lateral view; I1, upper view; I2, lateral view; J1, upper view; J2, lateral view. Each scale bar equals 100 μm.

More than 60 conodonts were obtained from three rock samples (Fig 2) collected from the mound base and the mound body of the uppermost first member and lower second member of the FXG Formation. The index *Hindeodus cf*. *parvus* was found in the microbialite of the mound base (Fig 7A–7F). *H*. *parvus* has been suggested as the index fossil of the base of the Triassic [58, 59]. However, because rock samples for conodont extraction were not collected at high sampling resolution, the first occurrence of the *H*. *parvus* could not be determined in relation to its first appearance datum, and thus, the Permian–Triassic boundary is difficult to identify. *H*. *parvus* can extend to *Isarcicella isarcica* zone [60, 61].

Furthermore, four conodonts from the microbial mound body were identified as *I*. *staeschei* (Fig 7G–7J), which is widely distributed in south China in the earliest Triassic. As a characteristic fossil of the lowermost FXG Formation in the eastern–north Sichuan Basin [62, 63], *I*. *staeschei* was recognized as the index fossil of the second Griesbachian conodont zone in several sections in south China (e.g., [64–66]); however, in other sections, the first occurrence of this species can be in the fourth Griesbachian conodont zone (e.g.,[63]).

**Fig 8:**
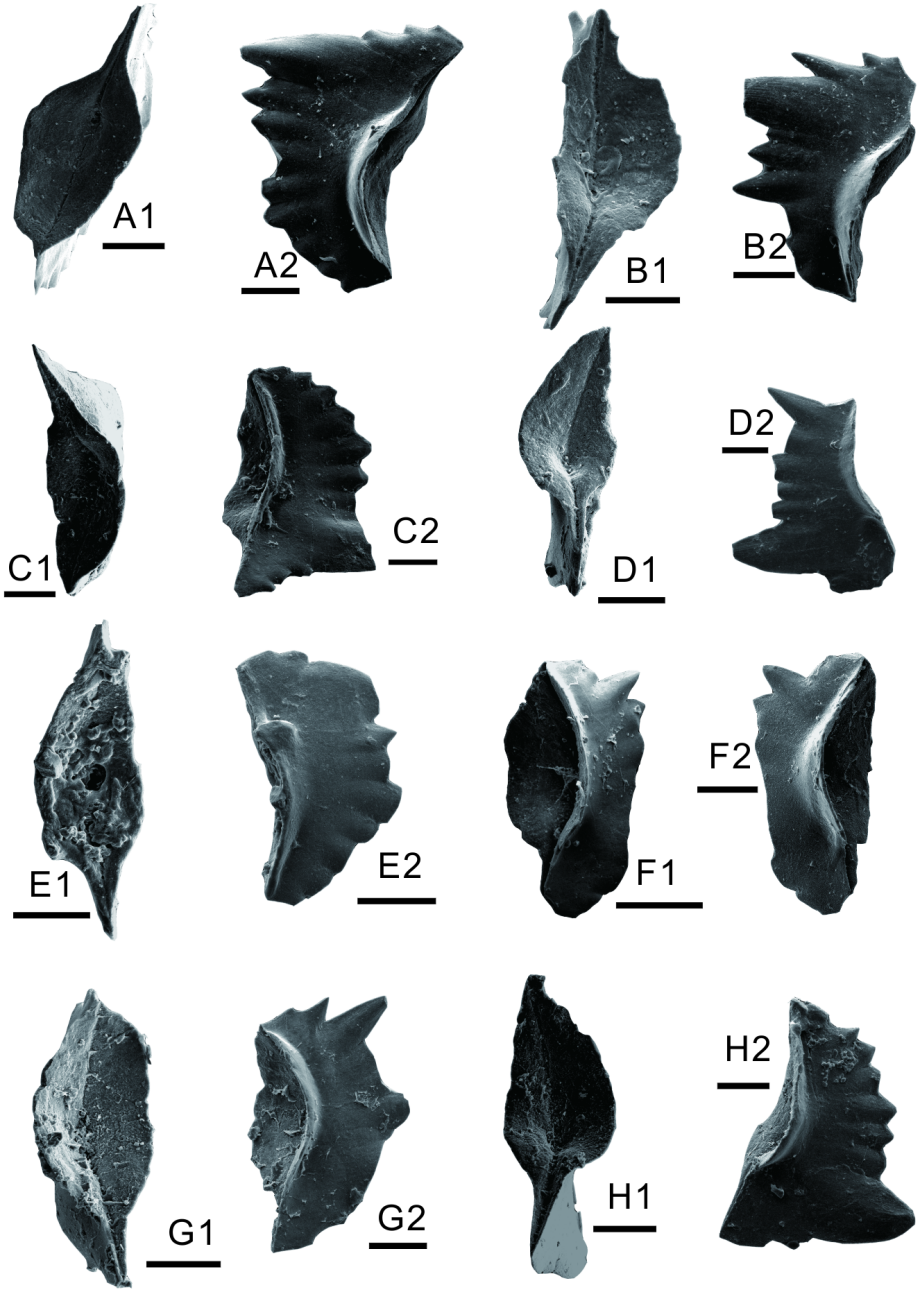
Conodonts from the lower second member of FXG Formation at Baimiaozi, Beibei. A, B, *Hindeodus n*. *sp*. *A*. A1, B1, aboral view; A2, B2, lateral view. C, D, E, F, G, *Hindeodus sp*. *Indeterminate*. C1, D1, E1, G1, aboral view; F1, oblique lower view; C2, D2, E2, F2, G2, lateral view. H, *Hindeodus parvus*? (Kozur and Pjatakova, 1976). H1, aboral view, H2, lateral view. Each scale bar equals 100 μm.

**Fig 9:**
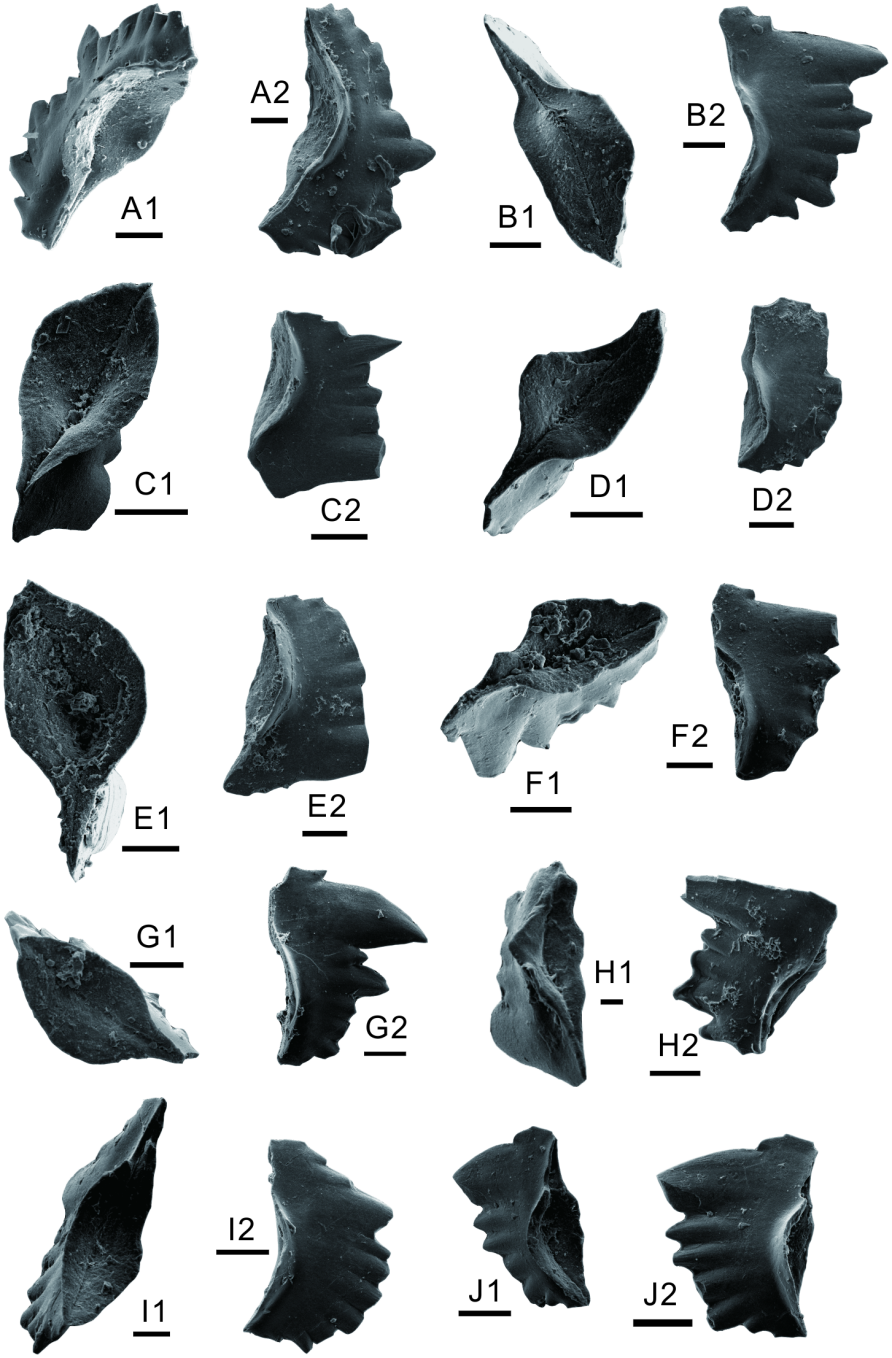
Conodonts from the lower second member of FXG Formation at Baimiaozi, Beibei. A, *Hindeodus cf*. *sosioensis* (Kozur, 1996). A1, oblique lower view; A2, lateral view. B, *Hindeodus n*. *sp*. *A*. B1, aboral view; B2, lateral view. C, D, E, F, I, J, *Hindeodus sp*. *Indeterminate*. C1, D1, E1, F1, I1, aboral view; J1, oblique lower view; C2, D2, E2, F2, I2, J2, lateral view. G, H, *Hindeodus parvus*? (Kozur and Pjatakova, 1976). G1, aboral view; H1, oblique lower view; G2, H2, lateral view. Each scale bar equals 100 μm.

Abundant conodonts were obtained from another sample of the lower part of the second member of the FXG Formation, about 24 m above the microbial mounds. This fauna is also dominated by *H*. *parvus*?, which is indicative of an early Griesbachian age (Figs 8 and 9).

In conclusion, the age of the microbial mounds can be characterized by the occurrences of *H*. *parvus*? and *I*. *staeschei*, and the exact horizon corresponds to the lower Griesbachian [58, 64, 65, 67]. The conodonts from the lower second member of the FXG Formation are dominated by *H*. *parvus*?, whose age is the early Griesbachian.

## 5. Discussion

### 5.1. Age of the first member of the FXG Formation at BMZ

At the BMZ Section, appearances of both *Hindeodus parvus*? and *Isarcicella staeschei* occur in Unit E, the uppermost part of the first member of the FXG Formation. *H*. *parvus*? was also found in Unit F of the lower second member of the FXG Formation, which indicates that the age of the lower second member of the FXG Formation is early Griesbachian. In the Shangsi Section in Sichuan, the *I*. *staeschei* zone is less than 7.0 m above the LPME line [63]. In total, units A–F in the BMZ Section is not less than 70.0 m. It may be attributed to the uncertainty of the conodont *Hindeodus parvus* in this interval. Another possible explanation for the dramatically thicker Griesbachian at BMZ is the duplication of strata caused by the reverse faulting. However, the fault displacement of the upper first member of the FXG Formation, which can be determined through the repeated lithological change from Unit E to Unit F, is <5.0 m. In the Beibei area, the massive oolitic limestone and the underlying grainstone are widely distributed, e.g., at the Laolongdong Section. Furthermore, the boundary of Units C and D that indicates a sudden lithological change from grainstone to oolite occurred, which suggests that there was no large displacement by the fault at the boundary of Units C and D (Fig 5L). Additionally, similar lower Triassic stratigraphy can be seen on the other side of the Jialing River, and the four members of the FXG Formation have an almost equal thickness with the studied section herein; thus, these features imply only a minor displacement effect by the two faults.

The tectonic, lithological, and stratigraphical investigation results revealed a greater thickness of the lower Griesbachian at BMZ, which suggests that there was a higher sedimentary rate in the study area, i.e., the Upper Yangtze Region. The high sedimentary rate of the lower Triassic has been widely verified, and it was likely the consequence of stronger land weathering and unusual oceanic chemical conditions shortly after the LPME (e.g.,[68]). Therefore, the mudstone, marl, and ooidal grainstone of Units A–D underlying the microbial mounds at BMZ reflect a rapid deposition. The distinct karst surface, which can be seen in the P–Tr transitional layers in the successive adjacent Late Permian to lower Triassic carbonate sections in the Chongqing area, was not found at the BMZ Section, possibly because of the lack of corresponding outcrops or because of the poorly defined P–Tr boundary. Thus it is difficult to determine whether the lower Griesbachian microbialites at Laolongdong are isochronal with the microbial mounds at BMZ.

### 5.2. Comparison of Griesbachian, Cambrian and Precambrian microbial mounds

Precambrian and Cambrian carbonate accretions formed by microbial communities, e.g., reefs and mounds, are primarily found in Australia, North America, China, and Africa [55, 56, 69–74]. Most of these microbial reefs or mounds have heights of about 50–100 m and base diameters of 100–200 m, and are commonly much larger than those at BMZ. Giant (>200 m thick; kilometers in diameter) dolostone mounds that accumulated on the floor of a restricted basin (the Borden Basin in Nunavut, Canada) are considered Precambrian carbonate seep mounds [75]. Mounds with sizes similar to those of the BMZ Section have been reported from other regions, e.g., the middle Cambrian domical mounds in western Wyoming (U.S.A.), and these are up to 1.0 m in height and 1.5 m in diameter at their base [76]. The Cambrian Series 2 microbial reefs of the North China Platform occur within a thin unit, ca. 3.0 m thick [73].

The large Precambrian and Cambrian microbial reefs and mounds commonly grew in low–moderate energy shelf areas. For example, the middle and late Early Cambrian microbial reefs in the central Transantarctic Mountains developed in such shelf areas [72]. The microbial reefs grew during a time of sea level rise and show a close relationship with the diapir [70]. However, the relatively low mounds developed in a depositional environment with low water energy. The Middle Cambrian algal mounds in western Wyoming and associated facies are similar to those found in modern shallow subtidal areas and lower intertidal flats [76]. The Cambrian microbial reefs in North China formed during the initial stage of a transgression and flourished in dynamic (tidal effects) and stressful (siliciclastic input) conditions [73]. However, the Precambrian giant carbonate seep mounds in Nunavut accumulated in deep-water regions of the Borden Basin, and these are limited geographically to the vicinity of basin-scale fault zones [75]. What is more remarkable is that some Cambrian carbonate accretions developed on facies deposited under high-energy circumstances, similar to the Early Triassic sequence of the BMZ Section. For example, thick ooidal grainstone units reflect deposition in high-energy shoals and as sand sheets associated with extensive microbial reef complexes [72]. In Wyoming, the Middle Cambrian microbial accretions are associated with intermound packstones containing ooids, large intraclasts, and skeletal debris [76].

The microstructures of the Precambrian and Cambrian microbial accretions differ from those of the BMZ Section. Stromatolites with irregular laminations are commonly seen in the Precambrian and Cambrian reefs and mounds (e.g., [56, 76]), whereas they were not observed at BMZ. The branching *Epiphyton* boundstone and *Hedstroemia* boundstone (e.g., [55, 69, 70, 73]) of the Precambrian and Cambrian microbial accretions, are not comparable with the early Griesbachian microbial mounds at BMZ, which probably implies that different environments existed for the marine calcareous microorganisms during these two periods. Previous studies [15, 77] indicated that the biological, geological events and environmental backgrounds of Precambrian-Cambrian and Permian-Triassic transitions were similar, e.g., CaCO_3_ supersaturation and depressed grazing. Theses paleo-oceanic conditions, which are different from that in modern ocean, resulted in the formation of microbial mounds during these two periods. Meanwhile, size of mounds was controlled mainly by the abundance of microbes, water depth and water energy of the sedimentary environment. As seen at BMZ, relatively prosperous microbe world stimulated the development of mounds in the photic zone and they were restricted in the deeper water with low energy.

### 5.3. Development of Early Triassic microbial mounds

Well-developed Early Triassic microbialites might indicate anomalous environmental conditions because modern microbialites tend to form in environments that are inhospitable to metazoans [78]. Microbial accretions in the Early Triassic developed under a harsh ocean environment [79, 80]. Such microbial accretions were distributed mainly on the eastern margin of Panthalassa and the periphery of the Paleo-Tethys Ocean, e.g., the Yangtze Region in southern China [31], western United States [51, 78, 81], southern Turkey [20], and eastern Greenland [32]. These mounds have similar sizes to those observed at BMZ, but are much smaller than the mounds of the Precambrian and Cambrian. According to a statistical analysis of the Early Triassic microbial accretions reported by Pruss and Bottjer [78], the developmental period of these microbial accretions featured two episodes, i.e., the Griesbachian and the Spathian. Sponges have been found in the Spathian microbial mounds, although none have been observed at BMZ [81]. Harsh marine environments, e.g., the high temperatures suggested by Sun et al.[82], during these two periods could have hindered the development of metazoans but stimulated the flourishing of microbes [78]. The extensive spatial distribution of the microbial mounds in the Triassic world possibly indicates a wide harsh marine environment, which would be in accord with the slow process of biotic recovery [78] in the Early Triassic.

**Fig 10:**
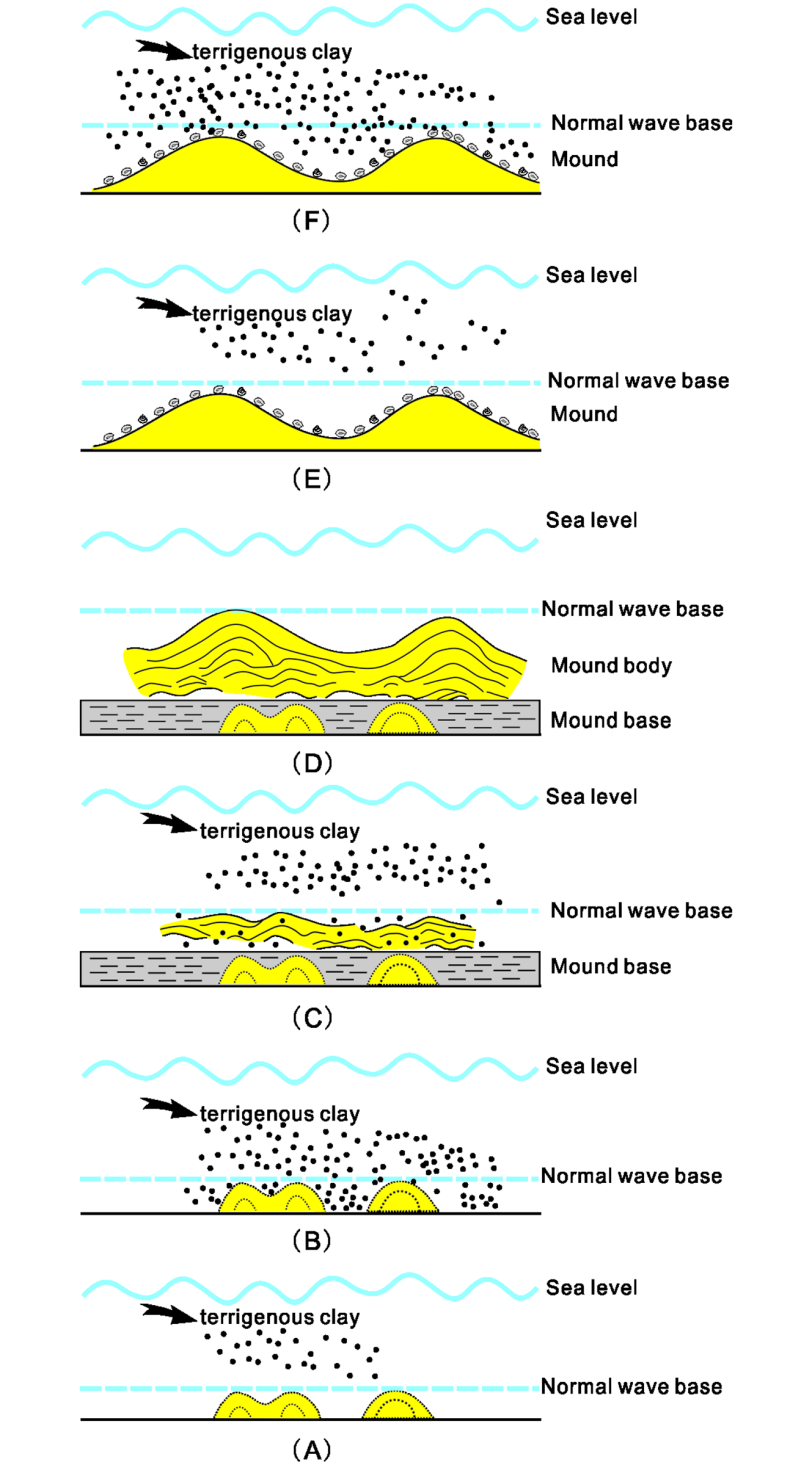
Model of the evolution of microbial mounds in the Upper Yangtze carbonate platform at Baimiaozi. (A) Decimeter-level microbial mounds initially grew at the base of the microbial mounds locations in a low-energy environment with low terrestrial input. (B) Massive terrestrial material transported to the shallow sea area, together with a rapid rise of sea level caused the failure of microbial growth. (C) In a normal shallow sea environment, the adequate supply of nutrients allowed microbes to flourish and microbial mounds became well developed. (D) A large number of microorganisms improved the Early Triassic marine environment via photosynthesis. (E)Metazoans of single species such as ostracods and gastropods that fed on the microorganisms thrived to a certain extent. (F) Rapid transgression caused hypoxia and superfluous inputs of terrigenous clay minerals into the deep water increased the turbidity; these events led to the demise of the microbial mounds. A large number of intact shells of dead organisms were found on the microbial mounds.

Microbialites bloomed in the shallow seas shortly after the LPME [20, 30], especially at Laolongdong in the Beibei area [30, 33, 41, 45, 83, 84]. However, to the best of our knowledge, microbial mounds are found only in the BMZ Section. The localized distribution of microbial mounds is interpreted as reflecting the coincidental sedimentary conditions in the Beibei area. The Upper Yangtze Block was located on the eastern side of the Paleo-Tethys Ocean and was near the equator [85]. The Early Triassic microbialites were distributed mainly on the carbonate platform [29, 38]. James [86] regarded the microbial mounds as quiet-water deposits that commonly grew below the wave base [87]. The karst surface developed at the uppermost Permian limestone at nearby sites such as Dongwan [39] and Laolongdong (e.g., [33, 41, 88]), and the overlying carbonate rocks of the Lowest Triassic are commonly microbialites that were deposited in shallow seas with relatively high energy, which restrained the growth of the microbial mounds. At BMZ, the Griesbachian sequence reflects a change of water environment from low (mudstone) to high (oolite) and then back to low energy (mudstone and marl of the second member of the FXG Formation). The microbial mounds developed as the water energy of the sedimentary environment decreased (Fig 10A). In addition, the sedimentary environment of these mounds were below normal wave base and in the lower part of euphotic zone. However, terrigenous clay inputs deposited in this low-energy environment could have restrained the development of carbonate petrogenic microbes. Thus, the decimeter-level microbial mounds, called aborted mounds in this study, developed in the base of the microbial mounds in lower Unit E (Fig 10).

The microbial mounds would have developed from the embryonic forms only if marine conditions permitted. The rate of continental weathering near the PTB increased dramatically in the Late Permian and lasted for about 2 Ma in the Early Triassic, which resulted in the deposition of a mass of weathered material in the shallow waters. Thus, clay concentrations generally increase in lower Triassic marine carbonate rocks [68, 89, 90]. A sharp increase in strontium isotopes recorded in the Early Triassic sequence in the Chongqing area also supports this hypothesis [46]. The input of weathered material would have increased the turbidity of the water of the seas, which would have had negative impacts on marine benthic organisms [68]. Furthermore, the global warming of the Early Triassic would have increased the frequency of stronger hurricanes and winter storms[91–93], thus disturbing nearshore sediments. Consequently, the abundance of nutrients necessary for microbial physiological activities, such as phosphate, would have contributed to the rapid flourishing of microorganisms [94]. In a normal shallow marine environment with lower contents of clay minerals, however, microbes would reach a certain degree of activity in response to the nutrients delivered by storms, and they could capture sediment for buildings mounds (Fig 10C and 10D). In addition, massive microbes constituted fodder for primary consumers such as shelly metazoan gastropods and bivalves, and their fossils were found enriched in the mound cap (Fig 10E). The demise of the mounds, as indicated by the lithological and environmental change at the boundary of the first and second members of the FXG Formation, could be attributable to rapid sea level rise and superfluous inputs of terrigenous clay minerals (Fig 10F).

Environmental conditions in the Early Triassic were poor, and the biotic recovery of the Triassic recovery was complicated [6, 95]. It is worth mentioning that the development of microbial mounds is indicative of the process of biotic recovery during the Early Triassic. These microbial mounds could be regarded as a fragile ecosystem that was mainly composed of microbes, and primary consumer such as ostracods and gastropods, characterized by high-abundance and low-diversity. During the earliest Triassic interval, the abundance of nutrients would had contributed to the rapid flourishing of microbes [30, 95, 96]. Microbes as the primary producers formed the foundation of the food web, and they not only improved maritime conditions by increasing the dissolved oxygen content of seawater through photosynthesis, but also constituted fodder for primary consumers such as shelly metazoan of gastropods and ostracods [95]. Besides, because of environmental improvements and adequate food, the shelly primary consumers could increase in number rapidly. However, at BMZ, the eventual elimination of the ecosystem dominated by microbes and metazoans was due to the change of the regional geological environment (e.g., rapid sea level rise and superfluous inputs of terrigenous clay minerals). Although the process of biotic recovery is complex and difficult to decipher, the development of these microbial mounds could provide a simple model for studying the process of biotic recovery in the Early Triassic.

## 6. Conclusions

1. This study investigated the Griesbachian microbial mounds found at BMZ in Beibei in the Upper Yangtze Region (southern China), which formed following the LPME. Field investigations and thin-section analyses indicated that the mound microbialites developed above the massive oolitic limestone of the carbonate platform of the first member of the FXG Formation. Three facies were identified from the microbial mounds, namely, a mound base, mound body, and mound cap. Irregular laminae were found in the microbialites of the mound base and the mound body. Light gray limestone of the mound cap contained intact fossils of ostracods, bivalves, and gastropods.
2. Conodonts from the mound microbialites in the uppermost first member of the FXG Formation and the overlying bioclastic limestone of the lower second member of the FXG Formation are indicative of an early Griesbachian age for the microbial mounds, and the findings suggest that the earliest microbial mounds in southern China developed shortly after the LPME.
3. The BMZ microbial mounds are considered to have grown at around the wave base, and the flourishing of the mounds was affected by changes in sea level, coastal storms that provided nutrients, and the relative clean sedimentary aqueous media. The demise of the mounds could be attributable to rapid sea level rise and superfluous inputs of terrigenous clay minerals. The development of microbial mounds is indicative of the process of biotic recovery during the Early Triassic.
